# Recombinant G-CSF-ApoAI Fusion Protein Is a Pleiotropic Factor

**DOI:** 10.3390/molecules31010119

**Published:** 2025-12-29

**Authors:** Svetlana Miroshnichenko, Mariya Pykhtina, Kirill Mosalev, Anatoly Beklemishev

**Affiliations:** Federal Research Center for Fundamental and Translational Medicine, 2 Timakova Street, Novosibirsk 630630, Russia; svmiro@yandex.ru (S.M.); mosalevkir@mail.ru (K.M.); ab.beklem-46@yandex.ru (A.B.)

**Keywords:** G-CSF-ApoAI, bone marrow cells, human MNCs, THP-1, cytokine expression, phagocytosis

## Abstract

In this study, we report the development of a recombinant human G-CSF fused with apolipoprotein A-I. The chimeric protein was expressed in *Pichia pastoris*. Using human bone marrow cells, the fusion protein was shown to retain the granulocyte activity of authentic G-CSF, more effectively inducing the differentiation and maturation of segmented neutrophils and maintaining the viability of progenitor cells. Using human mononuclear cells and THP cells, the resulting protein demonstrated monocytic activity, manifested by an increase in both total and CD14+ cell counts. By maintaining cell viability, the chimeric protein reduced the number of cells expressing caspase 3/7. G-CSF-ApoAI demonstrated accelerated cytokine regulation, promoting a more rapid transition of inflammation phases, accompanied by increased phagocytosis of latex particles, compared with G-CSF, increasing phagocytosis by 1.4-fold in the LPS-induced inflammation model. This suggests that this new pleotropic factor may be useful for pathogen clearance in infected wounds.

## 1. Introduction

Recombinant human G-CSF preparations are used in clinical practice for the treatment of neutropenia and autologous or allogeneic bone marrow transplantation. In addition, G-CSF is being studied for its potential use in the treatment of a number of other conditions not related to its hematopoietic functions, in particular in the treatment of wounds of various etiologies [1,2,3]. The ability of G-CSF to influence healing and resist infection has been confirmed in randomized clinical trials of diabetic wounds [4,5]. Growth factors play a crucial role in wound healing by controlling cell differentiation and proliferation, cell polarization, extracellular matrix remodeling, and wound maturation [6]. G-CSF stimulates the anti-infective defense of neutrophils, while the destructive potential of monocytes and lymphocytes is limited [7]. Later, it was shown that G-CSF polarizes the balance within T cell subpopulations from TH1 to TH2 [8]. Saito et al. found that G-CSF directly affects monocytes and modulates LPS-induced cytokine secretion in them [9]. Thus, G-CSF has broad therapeutic potential for clinical practice, beyond its hemostimulatory functions.

One of the important problems of using recombinant cytokines in therapy is their rapid degradation in the body; therefore, various strategies are used to prolong their action. In particular, chimeric forms of proteins were obtained in which the cytokine is fused with long-circulating plasma proteins such as albumin [10,11], transferrin [12], and immunoglobulin [13,14]. ApoA-I has promise as a partner protein in the creation of therapeutic fusion proteins, since in addition to the transport of various macromolecules, ApoA-I performs many regulatory functions in the body. In particular, ApoA-I exhibits anti-inflammatory [15], immunomodulatory [16], and antioxidant properties [17]; it is also involved in stimulating the proliferation of endothelial progenitor cells [18], and maintains the survival of mesenchymal cells under stress conditions [19].

It has been shown that the creation of hybrid forms of cytokines with ApoA-I significantly modulates cytokine function, reducing hematotoxicity and enhancing the immunostimulating properties of proteins [20,21,22].

It is expected that the creation of a G-CSF chimera with ApoA-I will combine the immunological activity of G-CSF with the biological benefits of ApoA-I. The aim of this study was to investigate the hematostimulatory properties of the new G-CSF-ApoA-I chimeric protein.

## 2. Results

### 2.1. Creation of the Recombinant P. pastoris Strain Capable of Producing rhG-CSF-ApoA-I

**Figure 1:**
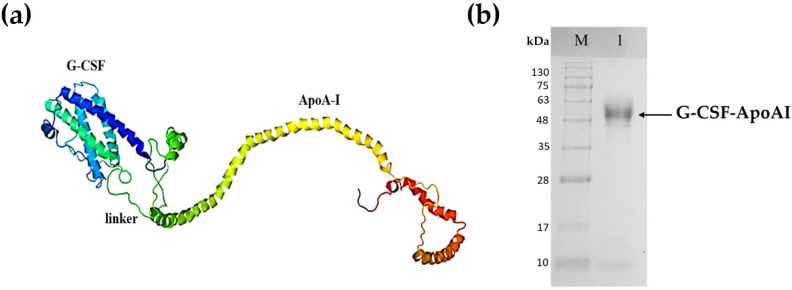
Recombinant chimeric protein G-CSF-ApoA-I. (**a**) Tertiary structure of G-CSF-ApoA-I; (**b**) SDS-PAGE analysis of purified recombinant chimeric G-CSF-ApoA-I. Lane M—standard protein molecular weight marker (Sib Enzyme) (10–200 kDa); 1—purified recombinant G-CSF-ApoAI.

A strain producing the G-CSF-ApoAI chimeric protein was obtained in the *P. pastoris* expression system. The calculated 3D structure of the chimeric protein is shown in Figure 1a. Cultivation of the recombinant yeast strain was carried out in an orbital shaker. The protein yield was low and amounted to only 5 mg/L of culture. The chimera was purified via two-step ion exchange chromatography on DEAE-Sepharose FF resin. The purity of the final preparation of the chimera was about 90–92% (Figure 1b).

### 2.2. Analysis of the Biological Activity of G-CSF-ApoAI

#### 2.2.1. Flow Cytometry Analysis of Human Bone Marrow Cells

**Figure 2:**
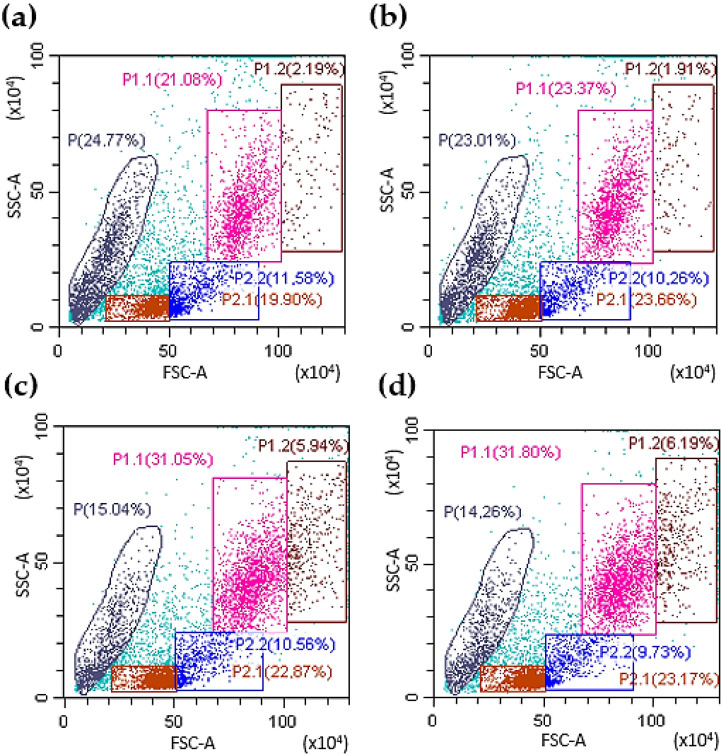
Representative FACS plots of BMC. The left panel (**a**) demonstrates the initial gating used for the bone marrow cells collected from controls using forward and side scatter (FSC/SSC), the right panel (**b**) demonstrates influence of ApoA-I on bone marrow cells. Panels (**c**,**d**) show the distribution of BMC in different gates under the influence of G-CSF and G-CSF-ApoAI (after 48 h of incubation). Gate P1.1 contains a population of mature granulocytes; P1.2—granulocytic progenitor cells; P2.1—lymphocytes, oxyphilic, polychromatophilic normocytes); P2.2 contains mainly monocytic cells and blast cells without granules (lymphoblasts, monoblasts). P contains the cell debris and smallest number of BMCs.

The effect of chimeric G-CSF-ApoAI and G-CSF on human bone marrow cells (BMCs) was initially analyzed via flow cytometry. Cells types were gated on the basis of cell size and granularity (FSC/SSC). Cells of the granulocyte series were defined as gate P1.1 and P1.2. Each gate located in the right region contains cells less mature than those located to the left. This division is indeed relative, but it can be verified by examining the cell cycle at each gate (Supplementary Materials Figure S1). It was found that both G-CSF-ApoAI and G-CSF had approximately the same activity on cells of the granulocyte series, resulting in an increase in the number of these cells in about 1.5 times compared with the control after 48 h of incubation (in control—21.1 ± 2.3%, under the influence of G-CSF and G-CSF-ApoAI—31.05 ± 2.1 and 31.8 ± 2.3%, respectively) (Figure 2). ApoA-I taken in an amount of 5 µg/mL did not have a statistically significant difference from the control (Figure 2b).

There is not a very significant difference in percentage between the effect of G-CSF and G-CSF-ApoAI on the granulocyte lineage of the BMCs. It should be noted that the number of cells of both the granulocyte series and other cells of the human BMC treated with chimera increased more significantly (1278 ± 31 cell/μL) than in control (865 ± 24 cell/μL) and G-CSF (1117 ± 28 cell/μL) at 24 h incubation. In addition, the number of progenitors under the action of G-CSF-ApoAI was slightly higher than in the case of G-CSF (Figure 2c,d, gate P1.2). Growth factors increased the total number of living cells by about 10% compared to the control. Both factors reduced the amount of cellular debris (14–15%) compared to the control (24.7%) (Figure 2).

#### 2.2.2. Analysis of the Cellular Composition of the Granulocyte Series of Human BMC Treated with G-CSF and G-CSF-ApoAI

For a more complete understanding of the results obtained using flow cytometry, a detailed myelogram of a number of human BMC granulocytes under the influence of the chimeric cytokine and G-CSF was performed (Table 1).

A detailed analysis of the myelogram made it possible to more accurately see the differences in the influence of growth factors on the granulocytic BMCs. Analysis of stained smears of human BMC samples showed that in the control, after 24 h of incubation, cells of the granulocyte lineage accounted for 27% of the total BMC population. In samples treated with G-CSF and G-CSF-ApoAI, it accounted for 41.1% and 36%, respectively. G-CSF dramatically activates the proliferation of progenitor cells, increasing the number of granulocytic cells by 1.5 times compared to the control, while G-CSF-ApoAI—only 1.3 times after 24 h of incubation. The survival of granulocyte progenitor cells were prolongs significantly by G-CSF-ApoAI, while under the action of G-CSF their number even slightly decreases. It can be seen that the pool of early granulocytic cells (blasts, promyeloblasts, myelocytes) that ensure the maturation of granulocytes decreases from 25% (after 24 h) to 16.6% (after 48 h) in the control. Its slight decrease is observed under the action of G-CSF (from 13.4% to 12.3%) and a pronounced increase from 11.6% to 18.2% under the influence of G-CSF-ApoAI (Table 1).

**Figure 3:**
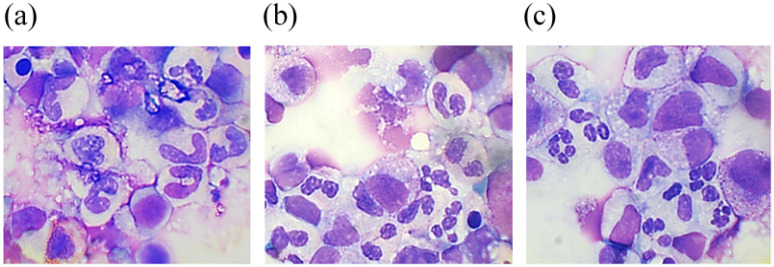
May–Grünwald and Giemsa-stained smears from a 48 h culture of bone marrow cells stimulated by G-CSF (**b**) and G-CSF-ApoAI (**c**) in comparison with the negative control (**a**).

A higher percentage of progenitor cells in the case of G-CSF-ApoAI according to myelogram (Table 1) readings corresponds to a higher cell count in S + G2/M phases when the study of the cell cycle is carried out via flow cytometry. Myelogram data showed that both cytokines promoted neutrophil maturation, reducing the number of abnormal hyposegmented neutrophils by 1.5-fold in the case of G-CSF and more than 2-fold in the case of G-CSF-ApoAI (Table 1, Figure 3). Notably, in the presence of the chimera, more efficient phagocytosis of the cellular debris was observed. This fact is of interest, for example, to study the possibility of using the chimeric protein in the therapy of wounds in patients with diabetes mellitus, who are characterized by the presence of impaired phagocytosis and neutrophilic mitosis.

#### 2.2.3. Study of the Proliferative Activity of Human BMCs Treated with G-CSF and G-CSF-ApoAI

Growth factors support the viability and proliferation of cells sensitive to their action. The study of the cell cycle allows an assessment to be made of the deposit of the factor to the proliferation of targeted cells. We conducted a study of the cell cycle of human bone marrow cells under the influence of G-CSF and G-CSF-ApoAI. Data on the cell cycle of all cells, as well as on the cell cycle of the granulocyte and monocyte gates, are presented in (Supplementary Materials Figure S1 II,III).

The gate in the right area on the flow cytometry histograms (FSC/SSC), as a rule, has an increased percentage and number of cells in the S + G2/M phases of the cell cycle. It can be seen that in the presence of the chimeric protein, the gates located to the right (P1.2) contain an increased number of cells in the S + G2/M active cell cycle phases (S-29.2%, G2/M-12%) compared to G-CSF (S-17.19%, G2/M-10.18%) and the control (S-16.2%, G2/M-10.1%).

**Figure 4:**
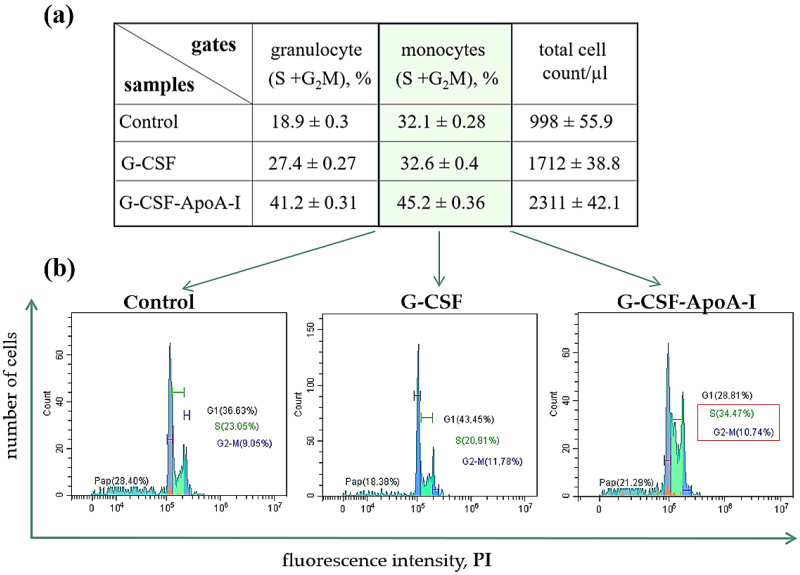
(**a**) Percentage of blast cells at S + G2/M cell cycle stages in P1.2 and P2.2 gates after 24 h incubation with G-CSF and G-CSF-ApoAI. (**b**) Flow cytometry cell cycle cytograms. Significant differences between groups were analyzed by the Mann–Whitney U test. Data are expressed as means ± standard errors; *p* ≤ 0.01.

The phases of the active cycle of the entire population of cells treated with the chimera were also higher (S-15.1%, G2/M-7.4%) than in the control (S-9.1%, G2/M-4.3%) (Supplementary Materials). The chimeric form containing ApoA-I stimulated the proliferation of the monocytes’ cells (Figure 4, Supplementary Materials Figure S1). This is an interesting fact, since we have previously shown that ApoA-I taken in an amount of 5–10 μg/mL stimulates the proliferation of the monocytic line of BMCs of rats [18]. In our study, G-CSF and the chimera were taken in the amount used for growth factors, that is, 50 ng/mL. The largest numbers of cells were in active cell cycle phases when treated with G-CSF-ApoAI and exceeded both control samples and BMCs treated with G-CSF (Supplementary Materials Figure S1).

Both factors supported the viability of the total number of BMCs. In the control, 30% of BMCs were apoptotic, while growth factors reduced this indicator by 1.7 times. A high proliferation rate and a decrease in apoptotic death contributed to an increase in the total number of cells in the presence of the chimera (Figure 4, Supplementary Materials Figure S1).

Thus, the obtained chimeric form exhibits the growth factor activity of granulocytes, and perhaps has additional activity against cells of the monocytic series. It can be said that G-CSF-ApoAI is a new growth factor with a broader spectrum of action than G-CSF.

#### 2.2.4. Study of the Monocyte-Stimulating Activity of G-CSF-ApoAI on Human MNCs

To better understand the effect of G-CSF-ApoAI on monocytic cell proliferation, human bone marrow mononuclear cells (MNCs) were isolated. MNCs do not contain granulocytes, allowing for a more accurate assessment of the effect of G-CSF-ApoAI on other KKM cells than those targeted by the granulocyte-derived factor. The effect of G-CSF-ApoAI on human MNCs was compared to both authentic G-CSF and GM-CSF.

**Figure 5:**
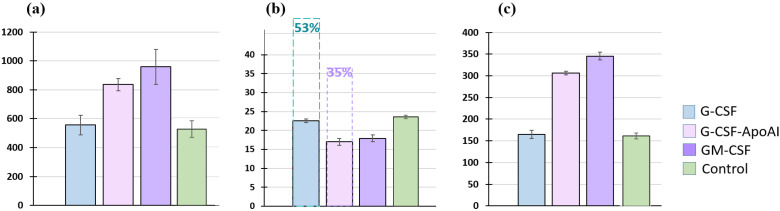
Representative histograms of the number of human MNCs after 24 h of incubation with the growth factors G-CSF, GM-CSF, G-CSF-ApoAI. (**a**) All cells (in μL); (**b**) caspases 3/7 (%). In pairs C/G-CSF-ApoAI, *p* = 0.0024, G-CSF/G-CSF-ApoAI, *p* = 0.0073, C/GM-CSF—*p* = 0.0075. Dotted line—caspase level after 48 h of incubation; (**c**) number of CD14+ cells. In pairs C/G-CSF-ApoAI, *p* = 0.0256; G-CSF/G-CSF-ApoAI, *p* = 0.0113; C/GM-CSF—*p* = 0.0245.

As Figure 5 shows, the incubation of human MNCs with G-CSF for 24 h did not significantly increase the total cell count or the number of CD14-expressing cells compared to the control (518 ± 56.76 cell/μL for control and 555.75 ± 67.57 cell/μL for G-CSF; for CD14+, 161.35 ± 6.85 cell/μL and 165 ± 9.43 cell/μL, respectively). Recombinant, GM-CSF and G-CSF-ApoAI equally increased both the total cell count and the number of CD14+ monocytes in the MNC culture (836.25 ± 42.5 cell/μL—G-CSF-ApoAI and 959.5 ± 121.1 cell/μL—GM-CSF), with CD14+ expression of 306.25 ± 4.64 and 345.27 ± 8.98 cell/μL, respectively, after 24 h of incubation (Figure 5a,c).

An analysis of the level of apoptosis was carried out using the expression of caspases 3/7 (Figure 5b). The percentage of cells expressing caspases was the same in the control and G-CSF at 24 h of incubation and was −23.7 ± 0.46% in the control samples and 22.57 ± 0.45% in the presence of G-CSF. Growth factors GM-CSF and G-CSF-ApoAI increased the level of cell survival, reducing the level of caspases to 17.9 ± 0.92% and 17 ± 0.89% for G-CSF-ApoAI at 24 h of cell culturing. At 48 h of incubation in the presence of G-CSF and in control samples, the level of caspases increased significantly (50–53%), while in the G-CSF-ApoAI and GM-CSF, their increase was less significant (Figure 5b).

Thus, it can be concluded that the stimulating effect of G-CSF-ApoAI on monocytic cells in MNC culture is similar to the effect exerted by GM-CSF, rather than G-CSF, i.e., the chimeric form of G-CSF containing ApoA-I affects the proliferation of not only granulocytes, as shown in Figure 2, but also CD14+ monocytes (Figure 5). Both factors—GM-CSF and G-CSF-ApoAI—maintained the viability of mononuclear cells throughout the experiment.

#### 2.2.5. Study of the Monocyte-Stimulating Activity of G-CSF-ApoAI the THP-1 Cell Line

**Figure 6:**
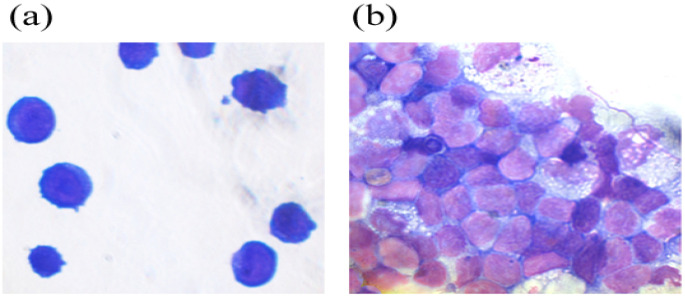
THP monocytic cells (**a**) and THP cells in the presence of the chimeric protein (**b**).

THP cells are a suspension line of monocyte-like cells expressing CD11b and capable of phagocytosis and antigen presentation. Depending on the environment, THP-1 can secrete pro- or anti-inflammatory cytokines. THP cells differentiate into monocytes expressing the LPS receptor, CD14+, in the presence of phorbol ester. The addition of G-CSF-ApoAI to the cells altered their morphology, with most cells becoming more similar to monocytes and macrophages (Figure 6).

**Figure 7:**
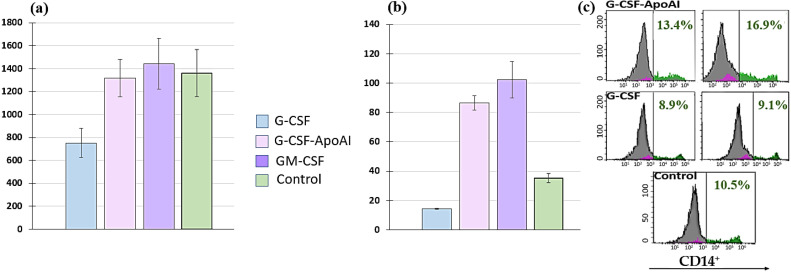
Representative histograms of the number of human THP cell lines after 24 h of incubation with growth factors G-CSF, GM-CSF, and G-CSF-ApoAI. (**a**) All cells (cells/μL). (**b**) CD14+ (cells/μL). (**c**) Representative histograms of flow cytometry measuring the number of CD14 cells. In pairs C/G-CSF-ApoAI; G-CSF/G-CSF-ApoAI; C/GM-CSF; G-CSF/GM-CSF—*p* = 0.000.

However, the number of CD14+ cells increased, but only slightly. The total number of cells in the presence of G-CSF was reduced by—752 ± 127.1 cells/μL, while in the presence of GM-CSF (1442.75 ± 219.3 cells/μL) and G-CSF-ApoAI (1316.5 ± 164.9 cells/μL), the total number was 1.8 times higher than G-CSF and close to the control values at 24 h of cell culturing (Figure 7a).

GM-CSF and G-CSF-ApoAI contributed to a statistically significant increase (*p* = 0.000) in the expression of CD14 on THP cells 102.3 ± 121.4 cells/μL and 87 ± 4.87 cells/μL, respectively (Figure 7b). In the presence of G-CSF-ApoAI, the number of CD14+ cells increased at 48 h of incubation, while no such increase was observed in the control and G-CSF (Figure 7c). Thus, G-CSF-ApoAI affects THP cells more closely than authentic G-CSF (Figure 7b). Based on these results, it can be hypothesized that the chimeric form of the G-CSF cytokine, containing ApoA-I, exhibits the properties of a pleiotropic factor, affecting several cell types and enhancing the proliferation of not only granulocytic cells but also monocytic cells. Similar values for CD14+ proliferation and differentiation in the presence of GM-CSF and G-CSF-ApoAI cytokines do not prove the identity of these growth factors. Therefore, cytokine expression under the influence of recombinant cytokines was further assessed in an LPS-induced inflammation model.

#### 2.2.6. The Effect of Recombinant Proteins on Cytokine Expression and Phagocytic Activity of Mouse Bone Marrow Cells

##### Evaluation of the Expression Level of Proinflammatory and Inflammatory Cytokines

A preliminary analysis of the mRNA profile of secreted cytokines in response to stimulation with the studied proteins in young mice (4 months) showed no differences, nor were there any differences in cell phagocytic activity. Therefore, in this experiment, we used BMCs from older mice (8 months).

**Figure 8:**
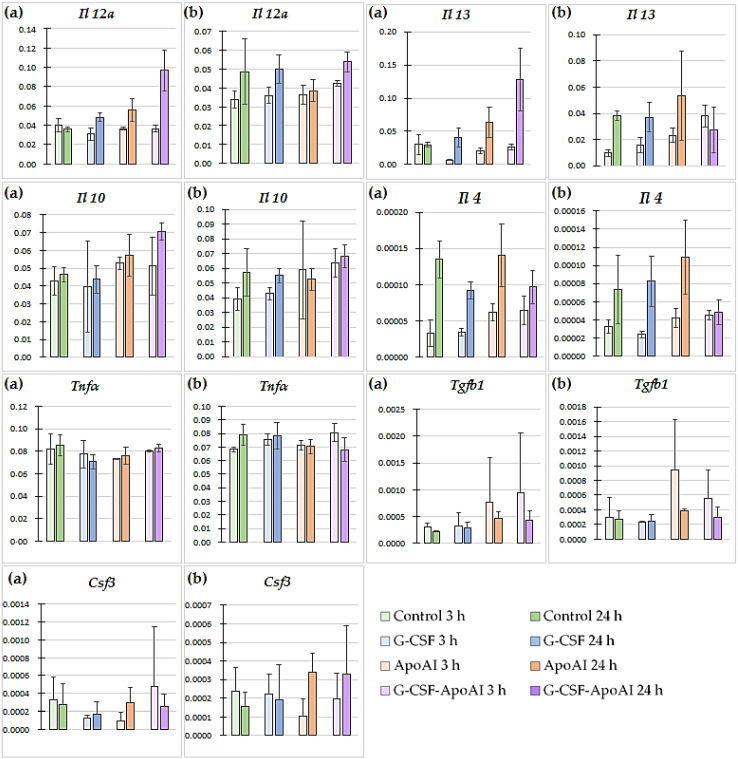
Cytokine gene expression levels in bone marrow cells of mice treated with the studied proteins (n = 3). (**a**) Gene expression in cultures without the addition of particles and LPS (no beads). (**b**) Gene expression in cultures in the presence of latex particles and LPS (bead). *p* values (Kruskal–Wallis test) for 3 h of the experiment (no beads; beads): *Il 12a*—0.43; 0.086, *Il 13*—0.066; 0.023, *Il 10*—0.59; 0.19, *Il 4*—0.059; 0.087, *Tnfa*—0.52; 0.072, *Tgfb1*—0.72; 0.46, *Csf3*—0.38; 0.54; for 24 h: *Il 12a*—0.029; 0.32, *Il 13*—0.038; 0.72, *Il 10*—0.066; 0.26, *Il 4*—0.17; 0.16, *Tnfa*—0.16; 0.49, *Tgfb1*—0.18; 0.31, *Csf3*—0.79; 0.29.

The expression of genes encoding IL-4, IL-10, IL-12α, IL-13, TGF-β, G-CSF, and TNF-α was studied using RT-PCR in bone marrow cells from mice cultured with recombinant proteins for 3 and 24 h. The analysis of differences in cytokine gene expression between the groups with and without latex particles is shown in Figure 8.

In the control group, the gene expression of all cytokines decreased 3 h after the addition of latex particles, and at 24 h, the mRNA levels of *Il 4*, *Tnfa*, and *Csf3* decreased, accompanied by an increase in those of *Il 12a* and *Il 10*. In the G-CSF and ApoA-I groups, no statistically significant changes in cytokine gene expression were observed under the influence of the particles, either 3 h or 24 h after treatment. Under the influence of G-CSF-ApoAI, 3 h after incubation with latex particles, an increase in the expression of the *Il 13* gene was observed (3.8-fold compared to the control, *p* = 0.0194) (Supplementary Materials Figure S2, Figure 8).

In the G-CSF-ApoAI group, in the presence of latex particles, after 24 h, a tendency toward a decrease in the expression of *Il 12* by 1.8 times and *Il 4* by 1.9 times was observed (*p* = 0.0808) compared to samples not stimulated with particles and LPS. After 24 h of incubation without particles, in a number of groups treated with G-CSF, ApoA-I, and the chimeric protein, an increase in the expression of the *Il 12* genes (1.3-fold, 1.6-fold, and 2.7-fold higher than the control, respectively, *p* = 0.029) and *Il 13* (1.4-fold, 2.13-fold, and 4.3-fold, respectively, higher than the control, *p* = 0.038) was observed (Supplementary Materials Figure S2, Figure 8a without particles). Thus, in the baseline state, in the presence of recombinant cytokines, the mRNA levels of both proinflammatory IL-12 and anti-inflammatory IL-13 were increased compared to the control. This increase was most significant in the chimeric protein group. The introduction of particles into the cell medium reduced the differences between the groups with the studied recombinant proteins and the control.

Time-dependent changes in cytokine gene expression were also observed within each experimental group with and without particles. The greatest difference in cytokine gene expression among the groups with the studied proteins was observed in the G-CSF-ApoAI group. Thus, IL-12 expression levels in the G-CSF-ApoAI group without particles were 0.036 ± 0.0034 at 3 h, and 2.7 times higher (0.097 ± 0.0021) after 24 h (Figure 8a). The introduction of particles caused a slight increase in *Il 12* expression at 3 h (0.042 ± 0.00542) compared to the group without latex particles, with a further insignificant increase to 0.0536 ± 0.00159 at 24 h of incubation (Figure 8). Thus, at 24 h of incubation, the expression level of the gene encoding IL-12 α was significantly lower in the group with particles compared to the group without particles in the presence of the chimeric protein (Supplementary Materials Figure S2). The most pronounced time-dependent changes were observed when studying the dynamics of *Il 13* gene expression. In the group without particles, its increase at 24 h in the G-CSF, ApoA-I, and G-CSF-ApoAI groups was 4-, 3-, and 5-fold, respectively (Figure 8). With the introduction of particles, the mRNA level of the gene encoding IL-13 increased over time by 4-fold in the control group and by 2.4–2.3-fold in the G-CSF and ApoA-I groups, respectively. At the same time, in the G-CSF-ApoAI group, there was a 1.4-fold decrease in *Il 13* gene expression by 24 h of incubation (0.0275 ± 0.00172) compared to at 3 h (0.038 ± 0.0084) (Figure 8, Supplementary Materials Figure S2).

The expression values of the *Il 12a* and *Il 13* genes in all groups without the introduction of particles at 24 h of incubation, according to the Kruskal–Wallis test, showed a statistically significant difference compared to the control (*p* = 0.029; *p* = 0.038, respectively). In the presence of latex particles, the expression of the *Il 13* gene showed a statistically significant increase in the series of G-CSF, ApoA-I, and G-CSF-ApoAI (*p* = 0.023, multiple comparison). A tendency towards an increase in the expression level of *Il 4* was observed from 3 to 24 h of incubation without latex particles in the control, G-CSF, ApoA-I, and G-CSF-ApoAI groups by 4, 2.6, 2.2, and 1.5 times, respectively. In the presence of latex particles, the dynamics of the increase in the expression level of *Il 4* was maintained in the C, G-CSF, and ApoA-I groups (2.2, 3.4, and 2.6 times) (Figure 8, Supplementary Materials Figure S2).

*Tgfb1* gene expression increased slightly only in the ApoA-I group at 3 h of incubation with latex particles (0.000942 ± 0.00006) (Figure 8), while *Tnfa* expression was increased at 24 h with particles only in the G-CSF group (0.0783 ± 0.01) (Figure 8, Supplementary Materials Figure S2). A slight, time-dependent increase in *Il 10* expression by 1.4 times was observed in the control in the presence of particles, while in the presence of G-CSF-ApoAI, the same difference was observed without latex particles.

All of the above time-dependent changes were found to be trending (*p* = 0.0808) according to pairwise comparisons, but they were confirmed by the results of multiple comparisons.

##### Evaluation of Phagocytic Activity of Mouse Bone Marrow Cells

**Figure 9:**
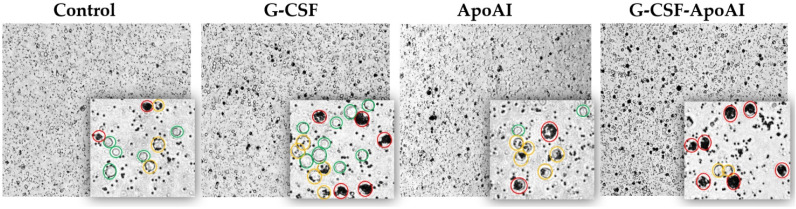
Mouse BMCs stimulated with LPS after incubation with polystyrene particles and the cytokines studied for 24 h of incubation. Cells are color-coded: complete phagocytosis (red), average phagocytosis (yellow), and absence of phagocytosis (green) (20×; 40×).

The level of phagocytosis of latex particles by mouse brain cells was assessed under the influence of the proteins G-CSF, G-CSF-ApoAI, and ApoAI, conditionally defining two groups of phagocytosis—complete phagocytosis and moderate phagocytosis (Figure 9).

**Figure 10:**
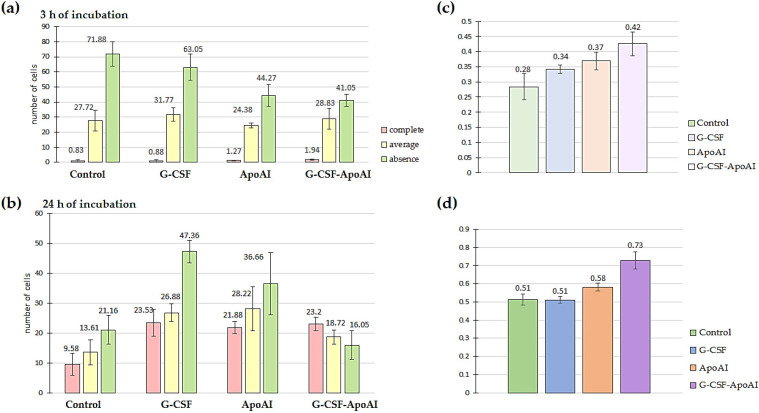
Evaluation of the phagocytic activity of LPS-stimulated mouse BMMCs after incubation with polystyrene and the studied cytokines for 3 h (**a**) and 24 h (**b**) of incubation. Figure (**c**,**d**) show the ratio of the number of phagocytic cells to the total number of mouse BMMCs for 3 h and 24 h of incubation with polystyrene particles and the studied cytokines; comparison of the proportions of phagocytosis (Z-criterion) for the control–G-CSF-ApoAI pair—*p* = 0.062 (**c**); Z-criterion for the control–G-CSF-ApoAI pair—*p* = 0.027 (**d**); for the G-CSF–G-CSF-ApoAI pair—*p* = 0.0088.

A qualitative examination of bone marrow cells using light microscopy revealed that at 3 h of incubation, the number of intensely phagocytic cells exposed to G-CSF-ApoAI was 2.2–2.33 times higher than in the control and G-CSF groups. Meanwhile, the number of non-phagocytic cells was minimal in the G-CSF-ApoAI group and maximal in the control group (Figure 10a). Overall, non-phagocytic cells predominated in all study groups at 3 h of incubation. It can be hypothesized that early activation of the *Il 12a*, *Il 10*, and *Il 13* cytokine genes promotes Th2 macrophage polarization and increased phagocytosis in the presence of the chimeric protein.

After 24 h of incubation of bone marrow cells with growth factors, the picture changed significantly. In all experimental groups, a tendency towards a sharp increase in phagocytic activity was observed compared to the control group. However, the total percentage of both intensively and moderately phagocytic cells was highest in the G-CSF-ApoAI group (72.3%), which was accompanied by a corresponding decrease in the number of cells not engaged in phagocytosis (16.05%), while in the presence of G-CSF, phagocytosis was 51.55% (Figure 10b). The Z-criterion showed a statistically significant difference between the G-CSF-ApoAI/control (*p* = 0.027) and G-CSF-ApoAI/G-CSF; (*p* = 0.0088) groups (Figure 10c,d). The difference in phagocytosis activation, which was evident at 3 h of incubation, became more pronounced by 24 h of incubation. The chimeric protein exhibited maximum phagocytic activity on mouse BMC, exceeding this indicator by 1.4 times compared to authentic G-CSF.

## 3. Discussion

G-CSF is a promising protein for use in a variety of pathologies; however, like most small-molecular-weight proteins, it has a short half-life. Protein fusion technology is one of the main approaches to extending the half-life of cytokines. This approach not only prolongs the action of cytokines in the blood but also reduces their acute phase of action and toxicity to the body. For example, G-CSF fusion proteins with IgG-Fc and IgG-CH not only had an increased half-life in vivo but also demonstrated improved hematopoietic properties [13]. Our previously obtained chimeric polypeptides GM-CSF-ApoAI and IFN-ApoAI demonstrated reduced cytotoxicity and new properties [23,24].

These data indicate that ApoA-I in the chimera imparts additional new properties to the fused cytokines, which may be useful for the treatment of a number of diseases. In this study, we investigated the properties of the newly obtained chimeric protein G-CSF-ApoAI. Since the creation of hybrid protein forms often leads to a decrease in the bioactivity of the cytokine [25,26], we first assessed the preservation of the granulocytic properties of the obtained chimeric protein. The ability of the chimera to induce a granulocytic link was studied in bone marrow cells, since bone marrow cells are a direct target of growth factors and cytokines. The bone marrow sample used in this study was characterized by a decrease in the number of granulocytic cells and impaired granulocyte maturation. The balance of differentiation was shifted towards younger forms of granulocytes. The chimeric form of the growth factor more effectively induced the maturation and differentiation of granulocytes, increasing the number of segmented neutrophils, decreasing the number of hyposegmented ones, and maintaining the viability of progenitors. Notably, the chimera also exhibited growth factor properties for other bone marrow cells, maintaining cellular diversity after 48 h of incubation. The new properties of the G-CSF-ApoAI chimeric protein, compared to G-CSF, are likely related to the modulating properties of ApoA-I. Previous studies have shown that ApoA-I, as a protein factor, regulates proliferation and maintains the stem cell properties of bone marrow progenitor cells under normal conditions, maintaining their viability under stress. In these studies, the working protein concentration was in the range of 5–40 μg/mL [19]. It is possible that, as part of the chimera, it exhibits these properties, supporting both the proliferation and viability of granulocytic and other bone marrow cell lines. In this case, the protein properties are manifested in the chimeric form, taken in nanogram quantities. In human mononuclear cells and the THP monocyte-like cell line, the chimeric form of the protein increased the number of CD14+ cells and reduced cell apoptosis, demonstrating the pleiotropic properties of the resulting protein, stimulating the proliferation and maturation of monocytic cells.

The stimulation of monocytic cell proliferation by G-CSF-ApoAI was likely due to the presence of ApoA-I in the chimera. ApoA-I is also capable of conjugating and neutralizing bacterial endotoxins [27]. The C-terminal region of ApoA-I, necessary for lipopolysaccharide binding, promotes complement-dependent bactericidal activity against the gastrointestinal pathogen, the Gram-negative bacterium Yersinia enterocolitica [28].

The ability of the chimeric protein to induce monocyte proliferation prompted us to further study this new function of the chimera and investigate its ability to enhance phagocytosis. Analysis of the phagocytic activity of the chimeric G-CSF in mouse bone marrow cells stimulated with LPS revealed the highest number of cells phagocytizing latex particles in the presence of the chimera, while G-CSF increased the total cell number, but the level of phagocytosis did not differ from control values. The analysis of the expression of a number of proinflammatory and inflammatory cytokines in mouse bone marrow cells exposed to the studied recombinant proteins revealed differences in the activation of cytokine profiles. Thus, in the presence of the ApoA-I protein, no activation of inflammatory cytokines was observed throughout the entire experiment, which is associated with the ability of the protein to bind LPS, which was used in our model to activate bone marrow cells. Cells stimulated by LPS in the presence of ApoA-I were not only stimulated by the endotoxin but also activated by ApoA-I into an anti-inflammatory state, since LPS was bound by the protein. G-CSF, however, stimulated inflammation, which persisted for 24 h, manifesting as an increase in TNF-α and a decrease in IL-10 after 24 h of incubation. This process was accompanied by an increase in the number of neutrophils stimulated by the cytokine. The first response to inflammation is the stimulation of neutrophil influx into the inflammatory site. G-CSF in the presence of LPS activates the proliferation of granulocyte lineages, increasing the cell count. The chimeric form, which contains G-CSF, activates inflammation at 3 h of incubation, similarly to G-CSF but more significantly, and sharply reduces the level of inflammatory cytokines at 24 h of incubation. The greatest increase in IL-12 in the presence of the chimera at 3 h of incubation activates macrophages, increasing inflammation. As is already known, IL-12 enhances the activation capacity of macrophages, which significantly enhances their phagocytic and bactericidal properties [29]. By promoting the rapid initiation of inflammatory cascades and enhancing the macrophage-mediated clearance of pathogens, IL-12 also exercises precise control over the magnitude and duration of inflammatory reactions [30]. Adeline Peignier et al. showed that S. Aureus infection strongly induces IL-12 production by monocytes [31]. While IL-13 induces the alternative activation of macrophages, thereby counteracting inflammatory processes caused by Th1 [32], stimulating the Th2 immune response reduces IL-12 production [33]. In our experiment, IL13 activated for 3 h promoted a decrease in IL12 for 24 h, while IL10 retained its anti-inflammatory effect for 24 h under the influence of the chimeric form. It was noted that IL-13 can effectively trigger the transition from the M1 to the M2a state [34] and, in the case of the chimeric cytokine, IL13 was probably the inducer of the triggering of Th2 polarization of macrophages phagocytizing latex particles. These accelerated dynamics of changes in IL-12 and IL-13 cytokine concentrations may be key in this case for redirecting cells toward phagocytosis. An initial experiment in young 4-month-old mice showed that BMC effectively phagocytized latex particles in the control group. It is worth emphasizing that the model was subsequently assembled to create conditions that specifically reduce the cell’s phagocytic function, as this problem is relevant for individuals with DFU.

Our data demonstrate the enhanced ability of the chimeric protein to regulate the cytokine profile, promoting a more rapid transition between inflammatory phases, accompanied by increased phagocytosis of latex particles. This suggests that this new pleiotropic factor will facilitate pathogen clearance in infected wounds.

## 4. Materials and Methods

### 4.1. Materials

All reagents for molecular biological research were purchased from Sigma-Aldrich (St. Louis, MO, USA) and Thermo Fisher Scientific Inc. (Waltham, MA, USA), and restriction endonucleases were purchased from SibEnzyme (Novosibirsk, Russia), DEAE Sepharose FF ion-exchange resin was purchased from GE Healthcare Bioscience (Uppsala, Sweden). RPMI 1640 media (Servicebio, Wuhan, Hubei Province, China), fetal bovine serum HyClone (South Logan, UT, USA), benzylpenicillin, and streptomycin were purchased from Gibco Thermo Fisher Scientific Inc., (Waltham, MA, USA), TrypLE Gibco Thermo Fisher Scientific Inc. (Waltham, MA, USA), APC-CY7 CD14 (BD Biosciences, Franklin Lakes, NJ, USA), Lymphocytes Separation Media density 1.077 g/mL (Capricorn Scientific, Ebsdorfergrunde, Germany), CellEvent™ Caspase-3/7 Green (Thermo Fisher Scientific Inc., Waltham, MA, USA), Propidium Iodide (Thermo Fisher Scientific Inc., Waltham, MA, USA), RNase (Thermo Fisher Scientific Inc, Waltham, MA, USA), LPS Sigma-Aldrich (St. Louis, MO, USA), latex for phagocytosis 1.5 μm, 10% suspension, Diaem, Novosibirsk, Russia).

#### Recombinant Proteins

Recombinant G-CSF and GM-CSF proteins obtained by biosynthesis in *Pichia pastoris* [23,35] were used as reference proteins in experiments studying the granulocyte and monocytic-cell activity of G-CSF-ApoAI. The ApoA-I was isolated from healthy blood donors by the method of sequential isolation of high-density lipoproteins (HDLs) by the standard method [36]. To analyze the phagocytic activity of G-CSF-ApoAI and study the expression of cytokines, the commercial G-CSF Granogen (Pharmpark, Moscow, RF) was used as a reference drug.

### 4.2. Methods

#### 4.2.1. Development of *P. pastoris* Strain Producing the Chimeric Protein G-CSF-ApoAI

The synthetic gene encoding a recombinant mature human G-CSF fused to ApoA-I was designed and optimized for expression in the yeast *P. pastoris*. The chimeric gene was cloned into the pPICZαA vector and expressed under control of the AOX promoter in *P. pastoris* X33. The selection of clones producing the chimeric protein was carried out on agar plates containing various concentrations of zeocin (500–2000 μg/mL). The recombinant strain was grown in BMGY medium in flasks on a shaker; chimera synthesis was induced with 1% methanol. The chimeric protein was purified using two-step ion exchange chromatography on DEAE-Sepharose FF resin under acidic and neutral conditions. Protein purity was analyzed by SDS-PAAG electrophoresis and by immunoblotting. Rabbit anti-ApoA-I IgG was used to detect the chimeric protein by immunoblotting.

#### 4.2.2. Bone Marrow Cell Isolation and Culture

(A) Human bone marrow aspirate was used to analyze the granulocyte activity of recombinant proteins. Human bone marrow was obtained with the voluntary consent of patients at the Institute of Clinical and Experimental Lymphology clinic ‘ICG SB RAS’ in accordance with the directives of the European Community (86/609/EEC) and the Helsinki Declaration and in compliance with the Ethical Principles for Scientific Medical Research with Human Participation No. 14 of 18 June 2019.

The bone marrow donor was a patient with a reduced number of granulocyte cells, delayed maturation, and segmentation abnormalities. The cell suspension was adjusted to a concentration of 2 × 106 cells/mL with RPMI medium containing 10% fetal bovine serum, and 0.5 mL was transferred to a 24-well plate. Bone marrow cells (BMCs) were cultured under standard conditions (37 °C, 5% CO_2_) for 24 and 48 h in the presence of recombinant cytokines (50 ng/mL) taken in equimolar quantities. Samples containing no additives were used as negative controls.

(B) Mouse bone marrow was used to analyze phagocytic activity and study the cytokine profile. Male C57BL6 mice (n = 3), 8 months old, were obtained from the vivarium of the Institute of Cytology and Genetics, Siberian Branch of the Russian Academy of Sciences. All animal procedures were carried out in accordance with protocols approved by the Biomedical Ethics Committee of the Federal Research Center of Fundamental and Translational Medicine (FRC FTM) (No. 31 of 21 November 2025) and following the recommendations for proper use and care of laboratory animals (European Communities Council Directive 86/609/CEE). Bone marrow cells (BMCs) were obtained under sterile conditions from mouse tibias and femurs using standard protocols. Red blood cells were lysed by suspending pelleted cells in 2 mL of lysis buffer (154.4 mM ammonium chloride, 10 mM potassium bicarbonate, and 97.3 μM EDTA tetrasodium salt) for 4 min. Then, RPMI was added and centrifuged (1400 rpm for 5 min). Subsequently, the cell suspension was adjusted to a concentration of 2 × 10^6^ cells per well with cell culture medium (RPMI, 10% fetal bovine serum (FBS)). Aliquots comprising 0.3 mL of the suspension were transferred to a 24-well plate and cultured in the presence of 5% db/db mouse serum (mimic type 2 diabetes). To assess phagocytosis and cytokine expression by mouse bone marrow cells under the influence of recombinant cytokines, a stock solution containing 1 ng/mL LPS and 0.05% latex particles was prepared. This solution was added to the wells to a final concentration of 0.03 ng/mL LPS and 0.015% latex particles. BMCs were cultured under standard conditions (37 °C, 5% CO_2_) for 3 h and 24 h in the presence of recombinant cytokines (150 ng/mL) taken in equimolar quantities. The experiment was carried out in triplicate. To determine the participation of the studied cytokines in the phagocytosis process, the expression level of inducible cytokines in the presence of latex particles was compared with the level of induced cytokines in a medium without particles.

#### 4.2.3. Isolation of Mononuclear Cells

The protocol for isolating mononuclear leukocytes from adult bone marrow was performed using standard centrifugation techniques using Ficoll–Paque (*p* = 1.077, Capricon, Ebsdorfergrunde, Germany). After 45 min of centrifugation at 300 *g*, mononuclear leukocytes remained directly above the Ficoll layer; MNCs are in the white layer (buffy coat) located between the PB plasma and the Ficoll, while red blood cells (>99%) settled to the bottom. MNCs were seeded in a 24-well plate at 1.2 × 10^6^ cells/mL. MNCs were cultured under standard conditions (37 °C, 5% CO_2_) for 24 h and 48 h in the presence of recombinant cytokines (50 ng/mL) taken in equimolar quantities.

#### 4.2.4. Culturing of THP Cell Line

Cells were cultured in RPMI-1640 medium with 10% fetal serum under standard cultivation condition (37 °C, 5% CO_2_, 20% O_2_ at humidified atmosphere). The cell density of the THP suspension culture was 1 × 10^6^ cells/mL. Cells were incubated for 24 and 48 h in the presence of recombinant cytokines (50 ng/mL) taken in equimolar quantities. Cells cultured without the addition of cytokines were used as a control. The analysis was performed on a CYTO FLEXS-100 flow cell. The total cell count and the number of CD14+ monocytes were measured.

#### 4.2.5. Flow Cytometry

BMCs were analyzed via flow cytometry (CYTOFLEXS-100, Beckman Coulter, Brea, CA, USA) as described previously [35]. Cells types were gated on the basis of cell size and granularity (FSC/SSC). Cell cycle were estimated using Propidium Iodide (PI) staining. The cells were harvested and fixed in ice-cold 70% ethanol for 2 h, and incubated for 30 min in a hypotonic solution of propidium iodide with RNase. The content of CD14+ cells was analyzed, the level of apoptosis was assessed by the % content of cells expressing caspases 3 and 7. Staining was performed according to the manufacturer’s instructions.

#### 4.2.6. Myelography

Bone marrow smears were prepared and stained with May–Grunwald and Giemsa dyes. Stained cells were assessed microscopically (Axio Scope A1 fluorescence microscope (Zeiss), Jena, Germany), and 500 BMCs per smear were counted.

#### 4.2.7. Cytokine Expression

RNA extraction from the biological material and reverse transcription were performed using Biolabmix reagents according to the manufacturer’s protocol. The relative amount of mRNA of the *Il 4*, *Il 10*, *Il 12a*, *Il 13*, *Tgfb1*, *Csf3*, and *Tnfa* genes was determined by quantitative real-time PCR using the BioMaster HS-qPCR SYBR Blue (2×) kit (Biolabmix, Novosibirsk, Russia). PCR conditions were optimized, and the best annealing temperatures were selected for each pair of primers (Table 2). Amplification was carried out under the following conditions: 95 °C—5 min; 45 cycles (95 °C—10 s, 15 s at the appropriate annealing temperature, 72 °C—15 s). To determine the expression level of the *Il 12a* gene, dimethyl sulfoxide (DMSO) was additionally added to the reaction mixture at a final concentration of 5%. To determine the expression level of the *Il 10* and *Il 13* genes, DMSO was additionally added to the reaction mixture at a final concentration of 2% to prevent the formation of amplification byproducts. The gene encoding GAPDH was used as a reference. Relative gene expression levels were calculated using the 2^−ΔCt^ method. Using this method, the efficiency of the polymerase chain reaction with real-time detection was calculated using the LinRegPCR version 2021.2 program. The calculated efficiency values were used to calculate the relative gene expression.

#### 4.2.8. Assessment of Phagocytic Activity

To assess the effect of recombinant cytokines on the phagocytosis of latex particles by mouse bone marrow cells (n = 3), the number of phagocytic and non-phagocytic cells was counted in four light fields. Each sample was examined in triplicate. Cell counting was performed using an Axio Observer Z1 microscope (Zeiss, Jena, Germany) at ×20 and ×40 magnification.

#### 4.2.9. Statistics

Gene expression data are expressed as the mean of several independent experiments ± standard deviation (M ± m). The statistical significance of differences was assessed using the nonparametric Wilcoxon–Mann–Whitney test for paired comparisons. For multiple comparisons, the Kruskal–Wallis test was used. The Z-test was used to compare the proportions of cells in the populations of experimental groups. Results were considered statistically significant when the significance level of differences reached *p* < 0.05. In the case of 0.05 < *p* < 0.1, a tendency towards one or another change was observed. The significance of differences between the study groups was determined using the STATISTICA 12 software package (StatSoft Inc., Tulsa, OK, USA).

## 5. Conclusions

Thus, the new chimeric G-CSF-ApoAI obtained through fusion technology possesses the properties of both an authentic colony-stimulating factor capable of supporting granulocyte proliferation and viability, and its own novel properties, including monocyte activation, induction of phagocytosis, and a more rapid reduction in inflammatory cytokine expression.

## 6. Limitation of the Study

A limitation of the study of the drug effects on cytokine gene expression in bone marrow cells was the significant cellular diversity of the bone marrow and the small sample size (n = 3) of animals for analysis, which forced us to use strict nonparametric statistical criteria. However, we did observe a dynamic change in cytokine gene expression, which can be confirmed in a larger sample using simpler study models.

## Figures and Tables

**Table 1:** Distribution of granulocytic cells of human BMCs treated with G-CSF-ApoAI and G-CSF compared to the control after 24 h and 48 h of incubation.

Cell Maturation Phases, (%)	24 h of Incubation			48 h of Incubation		
	Control	G-CSF	G-CSF-ApoAI	Control	G-CSF	G-CSF-ApoAI
blast/promyeloblast	8.9 ± 0.2	3.2 ± 0.15	3.2 ± 0.2	3.4 ± 0.2	3.5 ± 0.15	4.7 ± 0.15
myelocyte	16.1 ± 0.25	10.2 ± 0.25	8.4 ± 0.22	13.2 ± 0.25	8.8 ± 0.22	13.5 ± 0.25
metamyelocyte	13.4 ± 0.33	8.0 ± 0.22	7.3 ± 0.2	11.1 ± 0.27	6.6 ± 0.2	9.8 ± 0.2
band neutrophil	11 ± 0.5	5.6 ± 0.25	11.6 ± 0.3	19.1 ± 0.2	7.6 ± 0.2	6.6 ± 0.15
segmented neutrophil	21.3 ± 0.3	56.8 ± 0.4	58.1 ± 0.36	26.2 ± 0.25	57.1 ± 0.33	53.8 ± 0.3
segmentation anomalies	26 ± 0.35	16.4 ± 0.27	10.8 ± 0.25	27.3 ± 0.3	15.7 ± 0.2	11.1 ± 0.2
granulocyte content of all BMC, (%)	27 ± 3	41 ± 2.1	36 ± 2.6	20 ± 2.5	35 ± 2.7	34 ± 2.5

Data are expressed as means ± standard errors; *p* ≤ 0.05.

**Table 2:** Primer sequences.

Gene	Organism	Primer Sequences	Annealing Temperature
*Gapdh*	mouse	F: 5′-TAGACAAAATGGTGAAGGTCGG-3′ R: 5′-CCTGGAAGATGGTGATGGG-3′	57–64.5 °C
*Il* *12a*	mouse	F: 5′-AGTGTGGCACTGATGCTGATG-3′ R: 5′-GTAGCCAGGCAACTCTCGTT-3′	63.9 °C
*Il* *10*	mouse	F: 5′-TGGGTTGCCAAGCCTTATCG-3′ R: 5′-CTCTTCACCTGCTCCACTGC-3′	63 °C
*Il* *4*	mouse	F: 5′-TGAACGAGGTCACAGGAGAA-3′ R: 5′-CGAGCTCACTCTCTGTGGTG-3′	59 °C
*Il* *13*	mouse	F: 5′-TGTGTCTCTCCCTCTGACCC-3′ R: 5′-CACACTCCATACCATGCTGC-3′	64.5 °C
*Tnf*α	mouse	F: 5′-TGAGCACAGAAAGCATGATCC-3′ R: 5′-GGAACTTCTCATCCCTTTGGG-3′	60 °C
*Tgfb1*	mouse	F: 5′-TGATACGCCTGAGTGGCTGTCT-3′ R: 5′-CACAAGAGCAGTGAGCGCTGAA-3′	58 °C
*Csf3*	mouse	F: 5′-GAGCAGTTGTGTGCCACC-3′ R: 5′-CCAGCTGAAGCAAGTCCAAG-3′	58 °C

## Data Availability

All data are available within the manuscript and upon request to the corresponding authors.

## Supplementary Materials

The following supporting information can be downloaded at https://www.mdpi.com/article/10.3390/molecules31010119/s1. Figure S1: Distribution of BMC among gates after 24 h of incubation in the presence of growth factors in comparison with control. Figure S2: Expression levels of cytokines Il 4, Il 10, Il 12a, Il 13, Tgfb1, Csf3, and Tnfa by mouse bone marrow cells after 3 and 24 h of incubation with the studied proteins—G-CSF, G-CSF-ApoAI, and human ApoAI.
