# Supplementary material for: Recombinant G-CSF-ApoAI Fusion Protein Is a Pleiotropic Factor
Source: Molecules. 2025 Dec 29;31(1):119. doi: 10.3390/molecules31010119 (PMC12787894; doi:10.3390/molecules31010119)
Supplement: Supplementary file 1 [file molecules-31-00119-s001.zip › molecules-4043008-supplementary.pdf]

Supplementary Materials

Recombinant G-CSF-ApoAI Fusion Protein Is a Pleiotropic Factor

Svetlana Miroshnichenko, Mariya Pykhtina *, Kirill Mosalev and Anatoly Beklemishev

Federal Research Center for Fundamental and Translational Medicine, 2 Timakova Street, Novosibirsk 630630, Russia; svmiro@yandex.ru (S.M.); mosalevkir@mail.ru (K.M.); ab.beklem-46@yandex.ru (A.B.)

* Correspondence: pykhtina_maria@mail.ru

I. Control

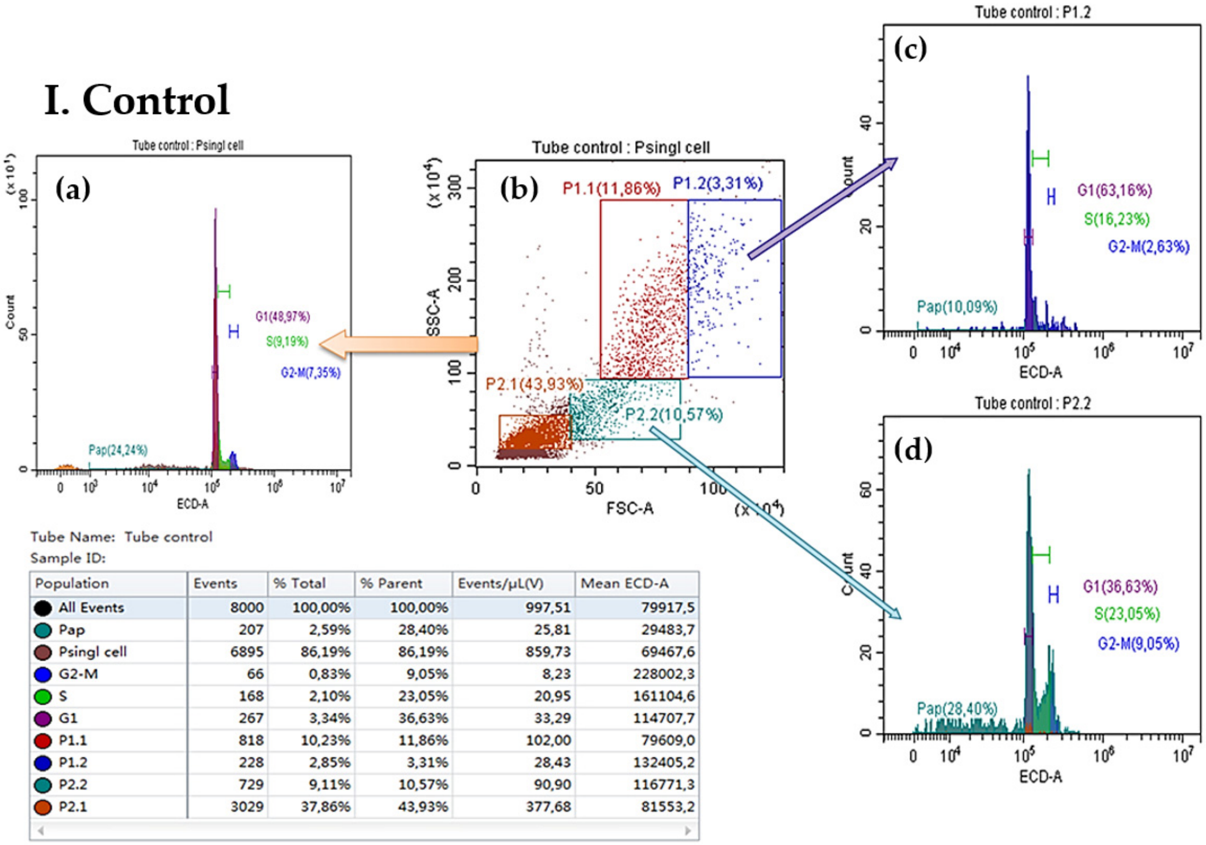

Tube Name: Tube control
Sample ID:

Population	Events	% Total	% Parent	Events/µL(V)	Mean ECD-A
All Events	8000	100,00%	100,00%	997,51	79917,5
Pap	207	2,59%	28,40%	25,81	29483,7
Psingl cell	6895	86,19%	86,19%	859,73	69467,6
G2-M	66	0,83%	9,05%	8,23	228002,3
S	168	2,10%	23,05%	20,95	161104,6
G1	267	3,34%	36,63%	33,29	114707,7
P1.1	818	10,23%	11,86%	102,00	79609,0
P1.2	228	2,85%	3,31%	28,43	132405,2
P2.2	729	9,11%	10,57%	90,90	116771,3
P2.1	3029	37,86%	43,93%	377,68	81553,2

II. G-CSF

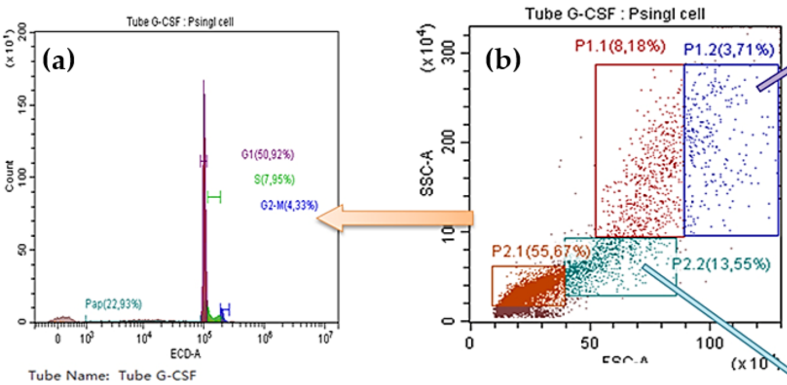

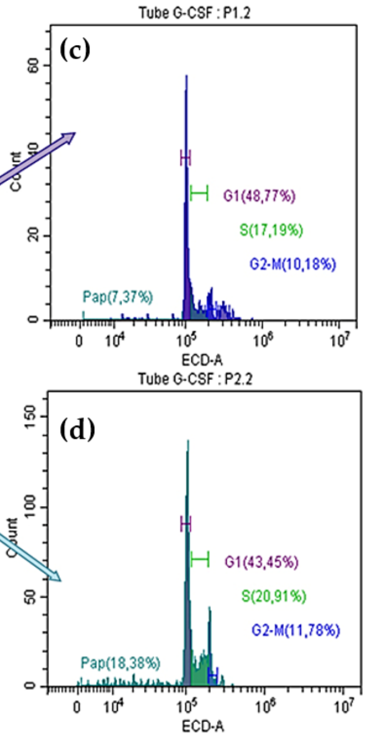

Tube Name: Tube G-CSF
Sample ID:

Population	Events	% Total	% Parent	Events/µL(V)	Mean ECD-A
All Events	8000	100,00%	100,00%	1711,94	80632,3
Pap	181	2,26%	18,38%	38,73	26515,2
Psingl cell	7270	90,88%	90,88%	1555,73	80723,7
G2-M	116	1,45%	11,78%	24,82	201638,3
S	206	2,58%	20,91%	44,08	149279,9
G1	428	5,35%	43,45%	91,59	101777,5
P1.1	595	7,44%	8,18%	127,33	83363,9
P1.2	270	3,38%	3,71%	57,78	148561,5
P2.2	985	12,31%	13,55%	210,78	114481,2
P2.1	4047	50,59%	55,67%	866,03	85277,7

III. G-CSF-ApoAI

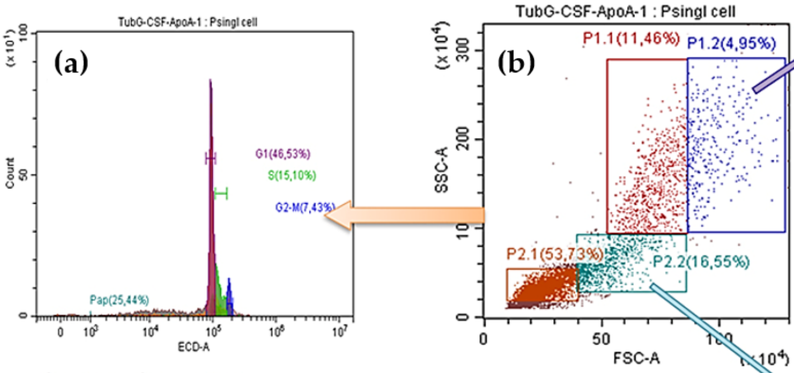

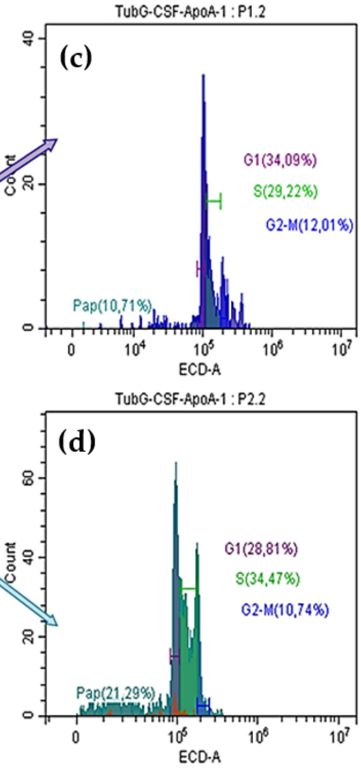

Tube Name: TubG-CSF-ApoA-1
Sample ID:

Population	Events	% Total	% Parent	Events/µL(V)	Mean ECD-A
All Events	8000	100,00%	100,00%	2311,38	79336,4
Pap	218	2,73%	21,29%	62,99	26780,1
Psingl cell	6187	77,34%	77,34%	1787,56	87639,3
G2-M	105	1,31%	10,25%	30,34	189256,5
S	327	4,09%	31,93%	94,48	140554,1
G1	333	4,16%	32,52%	96,21	97509,4
P1.1	709	8,86%	11,46%	204,85	79755,5
P1.2	306	3,83%	4,95%	88,41	133636,3
P2.2	1024	12,80%	16,55%	295,86	110246,8
P2.1	3324	41,55%	53,73%	960,38	86886,5

S1. Distribution of BMC among gates after 24 hours of incubation in the presence of growth factors in comparison with control. The right gate (P1.2, P2.2) contains the dividing cells. I - the control. The total number of cells is 997 cells/µL; II - G-CSF. The total number of cells is 1712 cells/µL; III - G-CSF-ApoAI. The total number of cells is 2130 cells/µL.

(a) - Cell cycle histogram of the distribution of the entire population of bone marrow cells by phases of the cell cycle. Pap -peak of hypodiploids, contains apoptotic cells;

(b) - Flow cytometry histogram of the distribution of BMC in coordinates (size) FSC /SSC (granularity);

(c), (d) - Cell cycle histograms for gates P1.2 and P2.2, respectively. The ordinate is the number of cells, the abscissa is the fluorescence intensity of propidium iodide.

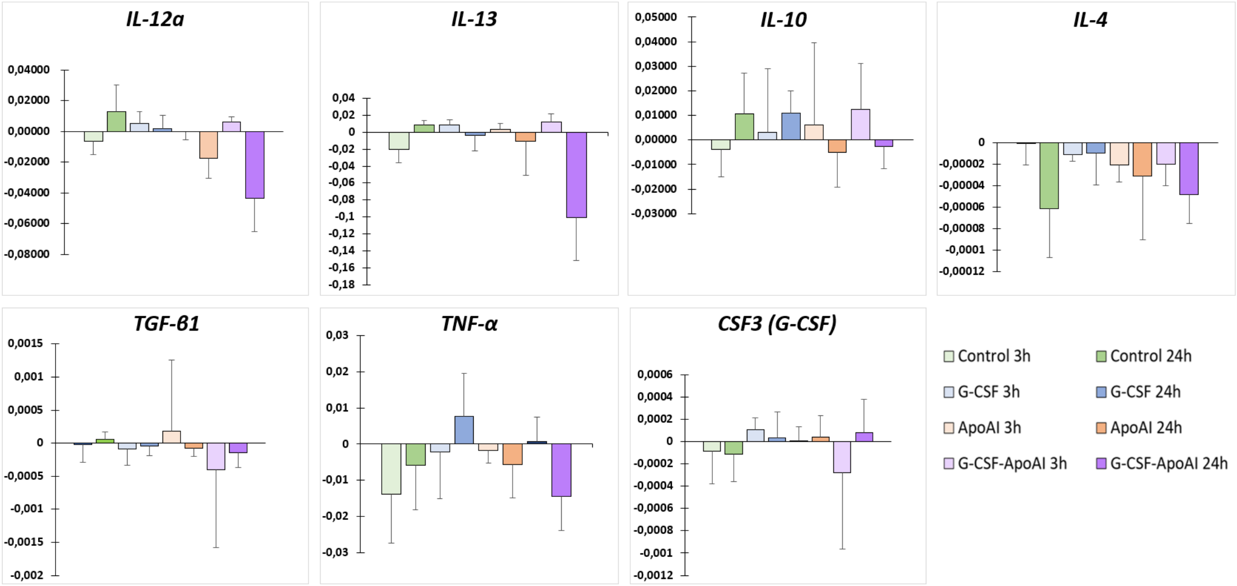

S2. Expression levels of cytokines *Il 4, Il 10, Il 12a, Il 13, Tgfb1, Csf3*, and *Tnfa* by mouse bone marrow cells after 3 and 24 hours of incubation with the studied proteins — G-CSF, G-CSF-ApoAI, and human ApoAI. The data are presented as the difference in cytokine expression in the presence of (LPS + latex beads) with the expression level in the absence of stimulation with this complex.
